# *CLDN3* inhibits cancer aggressiveness via Wnt-EMT signaling and is a potential prognostic biomarker for hepatocellular carcinoma

**DOI:** 10.18632/oncotarget.2288

**Published:** 2014-07-31

**Authors:** Lei Jiang, Yi-Dong Yang, Li Fu, Weiqi Xu, Dabin Liu, Qiaoyi Liang, Xiang Zhang, Lixia Xu, Xin-Yuan Guan, Bin Wu, Joseph J.Y. Sung, Jun Yu

**Affiliations:** ^1^ Institute of Digestive Disease and Department of Medicine and Therapeutics, State Key Laboratory of Digestive Disease, CUHK Shenzhen Research Institute, Li Ka Shing Institute of Health Sciences, The Chinese University of Hong Kong, Shatin, N.T., Hong Kong; ^2^ Department of Gastroenterology, The Third Affiliated Hospital, Sun Yat-sen University, Guangzhou, China; ^3^ Department of Clinical Oncology, The University of Hong Kong, Hong Kong, China

**Keywords:** CLDN3, hepatocellular carcinoma, metastasis, survival

## Abstract

Hepatocellular carcinoma (HCC) is one of the most common fatal malignancies but the molecular genetic basis of this disease remains unclear. By using genome-wide methylation profiling analysis, we identified *CLDN3* as an epigenetically regulated gene in cancer. Here, we investigated its function and clinical relevance in human HCC. *CLDN3* downregulation occurred in 87/114 (76.3%) of primary HCCs, where it was correlated significantly with shorter survival of HCC patients (P=0.021). Moreover, multivariate cyclooxygenase regression analysis showed that *CLDN3* was an independent prognostic factor for overall survival (P=0.014). Absent expression of *CLDN3* was also detected in 67% of HCC cell lines, which was significantly associated with its promoter hypermethylation. Ectopic expression of *CLDN3* in HCC cells could inhibit cell motility, cell invasiveness, and tumor formation in nude mice. Mechanistic investigations suggested through downregulation of *GSK3B*, *CTNNB1*, *SNAI2*, and *CDH2*, *CLDN3* could significantly suppress metastasis by inactivating the Wnt/β-catenin-epithelial mesenchymal transition (EMT) axis in HCC cells. Collectively, our findings demonstrated that *CLDN3* is an epigenetically silenced metastasis suppressor gene in HCC. A better understanding of the molecular mechanism of *CLDN3* in inhibiting liver cancer cell metastasis may lead to a more effective management of HCC patients with the inactivation of *CLDN3*.

## INTRODUCTION

Hepatocellular carcinoma (HCC) is the fifth most common malignancy in the world and the second leading cause of cancer death in Asia [1]. HCC is associated with multiple risk factors and is now recognized as a both genetic and epigenetic disease [2,3]. While the sequential accumulation of various genetic changes in hepatocarcinogenesis has been extensively studied, the contribution of epigenetic alterations to HCC development and progression has remained relatively poorly understood. Compelling evidence reveals that aberrant DNA methylation is a frequent event in HCC. Many studies showed that differentially methylated genes and CpG island methylator phenotype (CIMP) status in HCC were associated with clinicopathological features. Some commonly studied tumor suppressor genes (TSGs), such as *RASSF1A*, *p16*, *SOCS1*, *GSTP1* and *CDH1*, have been found to be hypermethylated in HCC in a cancer-specific manner, suggesting that aberrant DNA methylation is an essential incident for hepatocarcinogenesis [4].

Using methylated DNA immunoprecipitation coupled with DNA microarray (MeDIP-chip) analysis, we have identified that *CLDN3* was preferentially methylated in cancer [5]. *CLDN3* is located on Chromosome 7q11.23 and belongs to a family of proteins involved in the formation and function of tight junctions (TJs). It's well known that TJs function as the most topical barrier structure to fluid and cells in epithelial and endothelial cells. Disruption of TJs can trigger malignant transformation in a wide variety of epithelial cell types [6]. Early studies demonstrated that TJs were disorganized in HCC cells due to poor differentiation of the hepatocytes [7] and later studies found the disruption of TJ barrier could facilitate dissociated cancer cells to metastasize [8,9]. The backbones of TJs are mainly composed by occludins and claudins between adjacent cells. The dimerization of these trans-membrane proteins form a zipper-like structure to constrict the paracellular space to prevent solutes and water from passing through. The 24 members from human CLDN protein family are expressed in a tissue-specific pattern. Recent studies have reported CLDN family members in a wide range of human cancers and found these types of proteins are also expressed in a tumor-specific manner [10]. For instance, low expression of CLDN1 has been reported to be associated with progression and metastasis in breast and prostate cancer [11,12] while the elevated expression of CLDN1 exhibited structural and functional changes of epithelial to mesenchymal transition (EMT) –an important molecular event of metastasis [13]. Similarly, CLDN7 is highly expressed in renal cell carcinoma [14] but lost in invasive ductal carcinomas of the breast and in head and neck cancer [15,16]. These studies suggest the contribution of overexpression/loss of CLDNs in tumorigenesis may trigger different mechanisms and their expression pattern may be highly tissue-specific in different cancerous tissues.

In this study, we initially examined the expression level of *CLDN3* in human HCC cell lines and clinical HCC samples, as well as its correlation with promoter methylation status. Both *in vitro* and *in vivo* assays were used to study the tumor suppressive function of *CLDN3*. In addition, the tumor-suppressive mechanism of CLDN3 and its clinical significance in HCC was also investigated.

## RESULTS

### *CLDN3* is frequently downregulated in HCC

**Fig.1:**
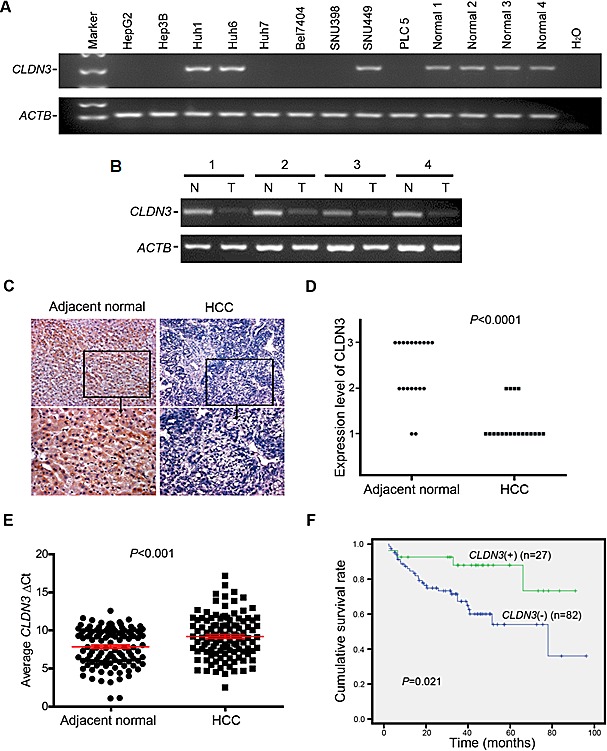
Downregulation of *CLDN3* in HCC (**A**) *CLDN3* expression was frequently down-regulated in HCC cell lines (A) and primary HCCs (cohort 1) (**B**) detected by RT-PCR. For primary HCCs, expression of *CLDN3* in tumor tissues (T) was compared with their paired nontumorous tissues (N). *ACTB* was used as a loading control. (**C**) Representative images of CLDN3 protein expression in a pair of HCC (*right*) and its adjacent normal tissue (*left*) determined by immunohistochemistry (IHC) with anti-CLDN3 antibody (*brown*). The slide was counterstained with hematoxylin. Original magnification, x200 (*upper*); x400 (*lower*). (**D**) CLDN3 protein expression level was indicated by IHC score according to the percentage of CLDN3 positive cells in primary HCC tumor tissues and their adjacent normal tissues (1,<20%; 2, 20-50%; 3,>50%). (*E*) *CLDN3* expression was frequently down-regulated in primary HCCs (cohort 2) detected by qPCR. Dot plots represent the ΔCt values of *CLDN3* (higher ΔC_T_ values correspond to lower expression; Mean ± SEM; Mann-Whitney U test). (**F**) Kaplan-Meier curves for overall survival rate of patients with HCC according to the expression level of *CLDN3*. *Green*, patients with normal *CLDN3* expression (n=27, mean survival time=78 months); *Blue*, patients with lower expression of *CLDN3* (n=82, mean survival time=60 months, P=0.021)

Semi-quantitative RT-PCR was initially used to study the mRNA expression status of *CLDN3* in 9 HCC cell lines, 4 normal human liver tissues, and 52 primary HCCs and their paired adjacent normal tissues (Cohort 1). *CLDN3* was significantly downregulated in 6/9 (66.7%) of HCC cell lines (HepG2, Hep3B, Huh7, Bel7404, SNU398, and PLC5) but was readily expressed in all 4 normal liver tissues (Fig. 1A). Expression of *CLDN3* was observed in all 52 tested adjacent normal tissues, whereas reduced expression of *CLDN3* was detected in 33/52 (63.5%) of primary HCCs (Fig. 1B). Protein expression level of CLDN3 was also studied by immunohistochemistry (IHC) staining in paraffin sections of 19 primary HCCs and their adjacent normal tissues obtained from Cohort 1 (Fig. 1C). Based on the score of staining, the average protein level of CLDN3 was significantly lower in HCC tumor tissues than that in adjacent normal tissues (1.2 vs. 2.4; P<0.0001; Fig.1D). Moreover, quantitative real-time PCR (qPCR) was performed to examine the mRNA expression level of *CLDN3* in 114 primary HCCs and their adjacent normal liver tissues (Cohort 2). Similarly, downregulation of *CLDN3* was detected in 87/114 (76.3%) of primary HCCs compared with their normal counterparts (defined as a 2-fold decrease of *CLDN3* expression in tumors) (Fig. 1E).

### *CLDN3* is an independent predictor of poor survival in HCC patients

The correlation between *CLDN3* expression status and clinicopathologic features of 114 HCCs was further evaluated, which was summarized in Table 1. The results showed that no correlation was observed between *CLDN3* downregulation and patient's gender, age, HbsAg, serum AFP, tumor size, cirrhosis, tumor cell differentiation, recurrence and tumor stage. However, Kaplan-Meier survival analysis showed that the overall 5-year survival rate was significantly lower in informative HCC patients with *CLDN3* downregulation (n=82, with a mean of 60 months) than that in HCC patients with normal *CLDN3* expression (n=27, with a mean of 78 months) (P=0.021, Fig. 1F). By univariable analysis, downregulation of *CLDN3* (P=0.029), presence of recurrence (P=0.003), poor differentiation (P=0.048), and advanced clinical stage (P=0.047) were significant negative prognostic factors for overall survival in HCC patients (Table 2). Nevertheless, multivariate analysis showed that downregulation of *CLDN3* (P=0.014) and recurrence (P=0.001) were two independent prognostic predictors for HCC patients (Cohort 2) enrolled in this study (Table 2).

**Table 1:** Association between CLDN3 mRNA expression and clinicopathologic characteristics of patients with HCC (*n* = 114)

Clinical features	Number	CLDN3 (-)	CLDN3 (+)	*P*-value
Gender Female Male	21 93	17 (81.0%) 70 (75.3%)	4 (19.0%) 23 (24.7%)	0.580
Age (yrs) <60 >60	97 17	73 (75.3%) 14 (82.4%)	24 (24.7%) 3 (17.6%)	0.526
HbsAg Negative Positive	18 90	16 (88.9%) 66 (73.3%)	2 (11.1%) 24 (26.7%)	0.159
Serum AFP (ng/ml) <400 >400	59 48	43 (72.9%) 37 (77.1%)	16 (27.1%) 11 (22.9%)	0.619
Tumor size (cm) <5 >5	48 62	39 (81.2%) 44 (71.0%)	9 (18.8%) 19 (29.0%)	0.214
Cirrhosis Absent Present	76 32	57 (75.0%) 24 (75.0%)	19 (25.0%) 8 (25.0%)	1.000
Differentiation Well differentiated (I) Moderately differentiated (II) Poorly differentiated (III-IV)	15 45 46	12 (80.0%) 34 (75.6%) 34 (73.9%)	3 (20.0%) 11 (24.4%) 12 (26.1%)	0.893
Recurrence or Metastasis Absent Present	57 54	46 (80.7%) 38 (70.4%)	11 (19.3%) 16 (29.6%)	0.205
TNM stage (AJCC) Early (I-II) Advanced (III-IV)	74 20	55 (74.3%) 14 (70.0%)	19 (25.7%) 6 (30.0%)	0.698

**Table 2:** Association of various factors with overall survival in 114 HCCs determined by COX regression model

	Univariate analysis		Multivariate analysis	
Variable	HR^a^(95%CI^b^)	*P*^c^	HR(95%CI)	*P*
Gender Male vs. Female	0.560(0.238-1.314)	0.183	-	-
Age ≤60yr vs. >60yr	0.987(0.958-1.017)	0.388	-	-
Tumor diameter ≤5cm vs. >5cm	1.146(0.560-2.343)	0.709	-	-
Cirrhosis Absent vs. Present	1.420(0.671-3.002)	0.359	-	-
Differentiation Well-Moderate vs. Poor	1.742(1.006-3.018)	0.048	1.785(0.981-3.248)	0.058
Recurrence Absent vs. Present	3.337(1.491-7.470)	0.003	5.431(2.034-14.503)	0.001
TNM stage Early vs. Advanced	2.315(1.013-5.290)	0.047	2.237(0.870-5.751)	0.095
CLDN3 expression Reduced vs. Normal	0.308(0.107-0.885)	0.029	0.247(0.082-0.749)	0.014

a HR: hazard ratio for death.

b CI: Confidence interval.

c P < .05 was considered statistically significant (in bold).

### Promoter methylation of *CLDN3* is correlated with its transcriptional inactivation

**Fig.2:**
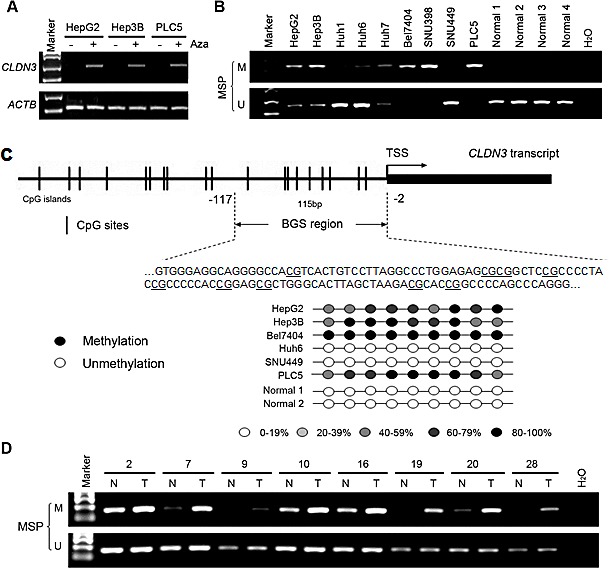
*CLDN3* promoter methylation status in HCC (**A**) *CLDN3* was restored after treatment with demethylating agent 5-aza-2'deoxycytidine (Aza) in three HCC cell lines without endogenous *CLDN3* expression. (**B**) Promoter methylation of *CLDN3* in HCC cell lines and normal liver tissues was determined by methylation specific PCR (MSP). (**C**) Representative high-resolution methylation profiles obtained by bisulfite sequencing of BGS region (-117 to -2), comprised of 9 CpG sites in the *CLDN3* promoter, in randomly selected 6 HCC cell lines and 2 normal liver tissues. For each sequenced DNA sample, the percentage methylation of each CpG was defined as the percentage of methylated CpGs from 6-8 randomly sequenced clones. Black circle, 80-100% methylated CpG; White circle, 0-19% methylated CpG; Light grey circle, 20-39% methylated CpG; Grey circle, 40-59% methylated CpG; Dark grey circle, 60-79% methylated CpG. (**D**) Representative MSP results of *CLDN3* in paired primary HCC (T) and adjacent normal (N) tissues.

To determine whether aberrant promoter methylation could contribute to downregulation of *CLDN3* in HCC, we treated 3 HCC cell lines with absent expression of *CLDN3* (HepG2, Hep3B, and PLC5) with the demethylating agent 5-Aza. The results showed expression of *CLDN3* was restored in all 3 HCC cell lines examined (Fig. 2A), suggesting that inactivation of *CLDN3* in HCC may be caused by its promoter methylation. The methylation status of *CLDN3* promoter was analyzed by MSP. Complete or partial methylation was detected in 6 HCC cell lines with absent expression of *CLDN3* (HepG2, Hep3B, Huh7, Bel7404, SNU398, and PLC5), whereas no or weak methylation was detected in other 3 HCC cell lines (Huh1, Huh6, and SNU449) and 4 normal liver tissues with *CLDN3* expression (Fig. 2B). To further explore the methylation details of *CLDN3* in HCC, 6 HCC cell lines with different degrees of *CLDN3* methylation were characterized by BGS. In line with the MSP results, the highest density of methylated CpG sites was found in Bel7404 and PLC5 cells with complete *CLDN3* methylation, whereas no methylated CpG sites was found in Huh6, SNU449 and normal liver tissues with strong *CLDN3* unmethylation (Fig. 2C). These results demonstrated that hypermethylation of the *CLDN3* promoter was associated with its transcriptional repression.

We next investigated the methylation frequency of *CLDN3* promoter in 30 primary HCC tumors and their paired adjacent normal tissues by MSP. Methylation of *CLDN3* was detected in 18/30 (60.0%) of the primary HCCs compared with their normal counterparts (Fig. 2D).

### Ectopic expression of *CLDN3* induces cobblestone-like morphology change and suppresses foci formation in HCC cells

**Fig.3:**
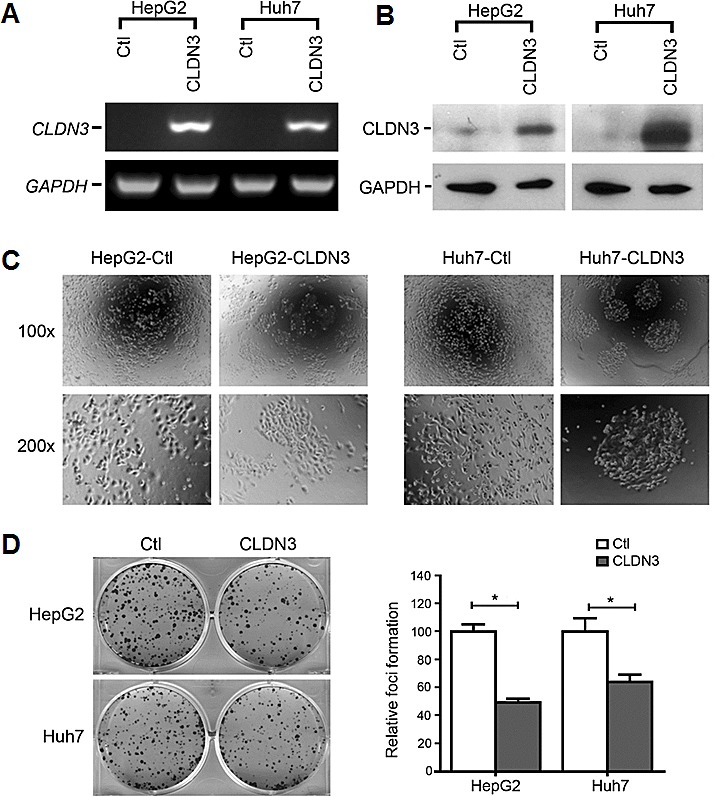
Morphological change and foci inhibition role of *CLDN3* in HCC cells Ectopic expression of *CLDN3* in HCC cell lines (HepG2 and Huh7) was confirmed by RT-PCR (**A**) and western blot (**B**). (**C**) Representatives of cell morphology of *CLDN3*-expressing cells (HepG2-CLDN3/Huh7-CLDN3) and control cells (*upper*, original magnification ×100; *lower*, ×200). (**D**) Representative of foci formation in monolayer culture. Quantitative analyses of foci numbers were shown in the right panel. Values were the mean ± SD of at least three independent experiments. *P<0.05; independent Student's *t*-test.

To determine if *CLDN3* has tumor-suppressive function, stably *CLDN3*-expressing clones were established from HepG2 and Huh7 cells. *CLDN3* gene and protein expression in these clones were confirmed by RT-PCR and Western blot analyses (Fig. 3A,B). The *CLDN3*-transfected HCC cells displayed an obvious morphological change compared with control cells. As shown in Fig. 3C, empty vector-transfected HCC cells grew in a normal state of monolayer culture while *CLDN3*-transfected cells formed cobblestone-like colonies (Fig. 3C). Ectopic expression of *CLDN3* in these HCC cells also caused a significant decrease in cell foci formation. The number of foci formed in *CLDN3*-transfected cells were significantly reduced than those in empty vector-transfected cells (down to 48%-63% of vector control, P<0.01, Fig. 3D). However, no obvious difference was observed between *CLDN3*- and empty vector-transfected HepG2 and Huh7 cells by cell proliferation, cell cycle and cell apoptosis analyses (Fig. S1, P>0.05).

### Metastasis suppressing ability of *CLDN3* in HCC cells

**Fig.4:**
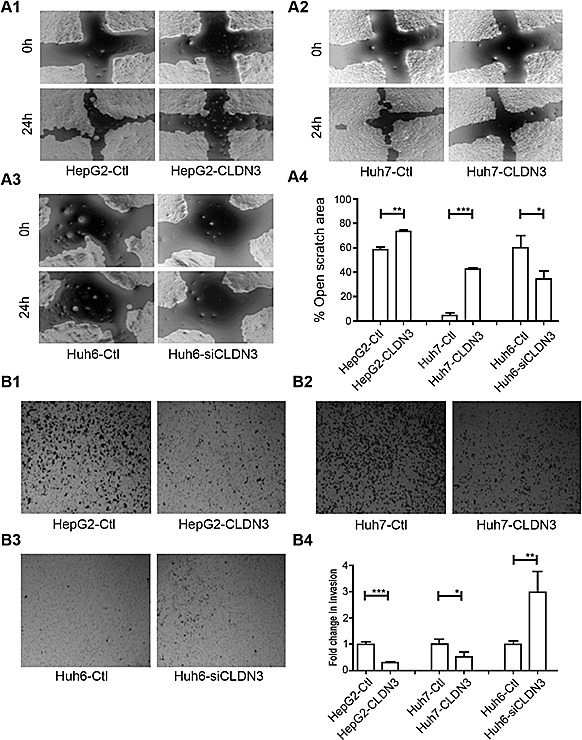
*CLDN3* inhibits cell mobility and invasiveness in HCC cells (**A1-3**) The effect of *CLDN3* on cell migration was determined by wound-healing assay. During a period of 24h, the spreading speed of *CLDN3*-expressing cells (HepG2-CLDN3/Huh7-CLDN3) along the wound edge was slower than that in control cells (HepG2-Ctl/Huh7-Ctl, **A1-2**); while knockdown of *CLDN3* in Huh6 cells by siRNA significantly promoted the cell mobility (**A3**). (**A4**) The percentage of open wound area was quantified in the right panel. (**B1-3**) Ectopic expression of *CLDN3* significantly inhibited the invasion ability in HepG2 (**B1**) and Huh7 (**B2**) cells; while knockdown of *CLDN3* by siRNA significantly promoted the invasion in Huh-6 cells (**B3**) by Boyden chamber matrigel invasion assay. (**B4**) The number of invaded tumor cells was quantified in the right panel. (*P<0.1, **P<0.05, ***P<0.01)

Since CLDN3 is a tight junction (TJ) protein and disruption of TJ barrier could facilitate dissociated cancer cells to metastasize [9], we thus determine the metastatic role of *CLDN3* in HCC cells by wound-healing assays and Boyden chamber matrigel invasion assays. Ectopic expression of CLDN3 could significantly suppress the migration in *CLDN3*-transfectants compared with control cells in HepG2 (58.7% ± 1.0% *vs.* 74.1% ± 0.4%, P<0.01) and in Huh7 (5.0% ± 0.8% *vs.* 42.1% ± 0.7%, P<0.001; Fig. 4A). Similarly, CLDN3 could also significantly inhibit the invasion in *CLDN3*-transfectants compared with control cells by invasion assays in HepG2 (29.8% ± 0.1% of control cells, P<0.001) and in Huh7 (52.2% ± 0.2% of control cells, P<0.05; Fig. 4B). To further confirm the metastasis suppressing ability of *CLDN3* in HCC cells, RNAi was used to knockdown endogenous *CLDN3* expression in Huh6 cells. The result showed that siRNA against *CLDN3* could significantly reduce *CLDN3* expression in Huh6 cells (Fig. S2). Twenty-four hours post siCLDN3 transfection, both wound-healing and invasion assays showed a significant increase of the cell migration (P<0.05, Fig. 4A3,4) and invasion ability (P<0.01, Fig. 4B3,4) in *CLDN3*-knockdown Huh6 cells compared with control cells.

### Ectopic expression of *CLDN3* inhibits tumor growth in nude mice

**Fig.5:**
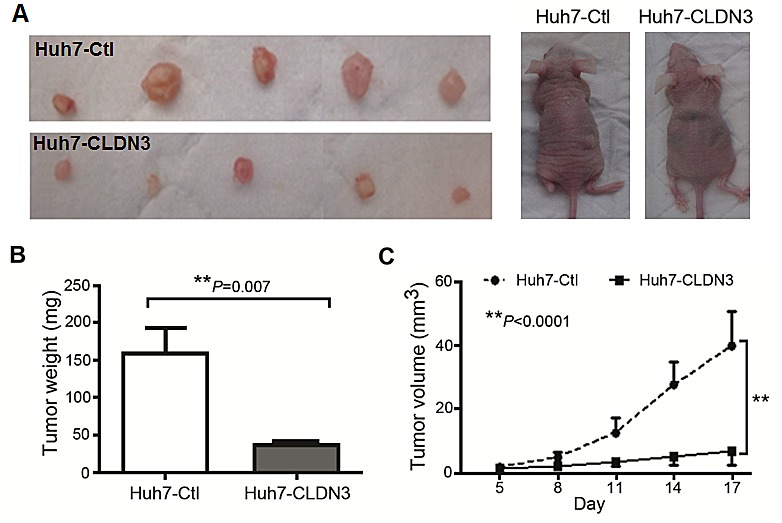
*In vivo* tumor suppressive role of *CLDN3* in HCC (**A**) Representative examples of tumors formed in nude mice following injection of *CLDN3*-expressing Huh7 cells (*lower panel*) and Huh7-Ctl cells (*upper panel*). (**B**) Histogram shows quantitative result of the tumor weight from Huh7-CLDN3 group and control group. Value are mean ± SD. P=0.007. (**C**) Tumor growth curves of *CLDN3*-expressing Huh7 cells (Huh7-CLDN3) in nude mice were compared with Huh7-Ctl cells by tumor xenograft experiment. The average tumor volume of Huh7-CLDN3 cells vs Huh7-Ctl cells was expressed as mean ± SD in 5 inoculated sites for each group of cells. **P<0.0001.

To further explore the *in vivo* tumor suppressive ability of *CLDN3*, tumor formation in nude mouse was tested by injection of Huh7-CLDN3 cells (n=5), while Huh7-Ctl cells (n=5) were used as controls. Within 17 days, solid tumors were readily visible in right hind legs of all 10 mice injected with Huh7-CLDN3 cells and Huh7-Ctl cells, respectively (Fig. 5A). The mean tumor weight was significantly less in Huh7-CLDN3 inoculated nude mice than those in Huh7-Ctl cells injected nude mice (159.2 ± 34.2mg vs. 36.6 ±10.9mg, P<0.01, Fig. 5B). Moreover, the size of tumors caused by Huh7-CLDN3 cells was significantly smaller than tumors induced by Huh7-Ctl cells (tumor volume: 40.0 ±11.9mm^3^ vs. 7.10 ±3.22mm^3^, P<0.0001, Fig. 5C). These results demonstrated that *CLDN3* has a strong tumor suppressive ability *in vivo*.

### *CLDN3* inactivates WNT–EMT signaling pathways in HCC cells

**Fig.6:**
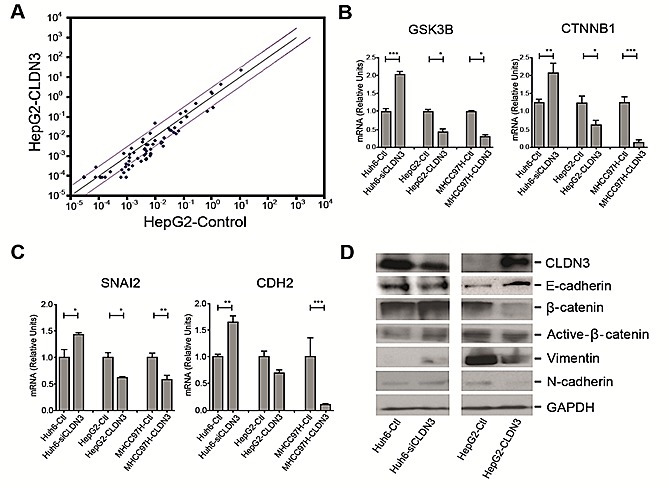
CLDN3 inactivates Wnt-EMT signaling in HCC cells (**A**) The scatter plot of PCR array analysis graphs the expression level (2^−ΔCt^) of genes related to Wnt signaling pathway in the HepG2-CLDN3 cells versus the HepG2-Ctl cells. The black line indicates fold changes (2^−ΔΔCt^) of 1. The pink lines indicate a fold change of 3 in gene expression. (**B, C**) Histograms represent the fold up or downregulation of *GSK3B* and *CTNNB1* (**B**), as well as *SNAI2* and *CDH2* (**C**) in CLDN3-overexpression or knockdown HCC cells by qPCR. (**D**) Expressions of epithelial markers E-cadherin, β-catenin, active β-catenin and mesenchymal markers Vimentin and N-cadherin were compared by Western blotting analysis between CLDN3-overexpression or knockdown HCC cells. GAPDH was used as loading control.

To explore the mechanism underlying metastasis inhibition by *CLDN3*, deregulated genes involved in Wnt signaling between *CLDN3*-transfected HepG2 cells and control cells were determined by Wnt signaling pathway PCR array. From the 84 Wnt pathway related genes, 5 were decreased (*GSK3B*, *CCDN1*, *CTNNB1*, *SLC9A3R1*, and *CSNK1D*) and one (*FZD3*) was increased significantly (fold change >3, P<0.05) in HepG2-CLDN3 cells compared with HepG2-Ctl cells (Fig. 6A). Two significantly downregulated key modulators of the canonical Wnt pathway *GSK3B* (-5.28 fold) and *CTNNB1* (-3.25 fold) were further verified by qPCR in *CLDN3*-transfected HepG2 and MHCC97H (a well-known metastatic HCC cell line), as well as in *CLDN3*-knockdown Huh6 cells (Fig. 6B). Furthermore, the correlation between *CLDN3* expression and Wnt pathway induced molecules related to epithelial-mesenchymal transition (EMT) were also examined by qPCR. Among the 6 genes tested (*CDH1*, *CDH2*, *CTNNB1*, *ZEB1*, *SNAI1*, and *SNAI2*), one EMT-inducing transcription factor (*SNAI2*) and one mesenchymal marker (*CDH2*) showed significant negative correlation with *CLDN3* expression in *CLDN3*- knockdown or overexpression HCC cells (Fig. 6C). The protein level change of EMT markers in *CLDN3*-knockdown/overexpression HCC cells were further confirmed by western blotting. Expression of E-cadherin (encoded by *CDH1*), β-catenin (encoded by *CTNNB1*), active β-catenin, mesenchymal marker N-cadherin (encoded by *CDH2*) and Slug (encoded by *SNAI2*) showed significant negative-correlation with CLDN3 expression in Huh6 and HepG2 cells (Fig. 6D), suggesting that *CLDN3* could act as a negative regulator of the WNT–EMT signaling pathways in hepatocarcinogenesis.

## DISCUSSION

CLDN3 belongs to a family of proteins important in tight junction formation and function. Recently, it has become apparent that *CLDN* gene expression is frequently altered in several human cancers [17], its expression pattern and biological function are largely unrevealed in human liver cancer. Here, for the first time, we showed that *CLDN3* was frequently downregulated in human liver cancer and its downregulation was significantly associated with poor survival of HCC patients. We found that reduced expression of *CLDN3* was caused by promoter hypermethylation in HCC cells. This is in line with previous studies demonstrating that the expression level of *CLDN3* was epigenetically regulated by promoter methylation in esophageal cancer [18] and ovarian cancer cells [19]. The expression of other claudin family members, such as *CLDN1* in podocyte [20] and breast cancer [21] and *CLDN4* in bladder cancer [22], has also been reported to be regulated through promoter hypermethylation.

It has been widely accepted that disruption of TJ barrier is a common event in various human cancers and loss of TJ function is correlated to cancer progression and metastasis [23]. Disassembly of TJs can cause loss of cell polarity and increased cell invasiveness [23, 24]. The claudin family proteins are known to form the “backbone” of TJs between cells and their expression profile in different tissues are highly tissue specific [25, 26]. *CLDN3* was uniformly expressed in the normal rat liver [27] and an increased expression of *CLDN3* was observed in liver regeneration 2-3 days after hepatectomy [28], suggesting the potentials of *CLDN3* in maintaining normal liver function and regeneration of fully polarized normal hepatocytes. In this study, we observed a cobblestone-like epithelial morphology and cellular aggregation in cultured *CLDN3-* overexpression HCC cells. In contrast, control HCC cells without endogenous *CLDN3* displayed a spindle shape, fibroblast-like mesenchymal morphology, and pronounced cellular scattering in monolayer culture, suggesting the inactivation of *CLDN3* in HCC might be related to EMT and cancer invasion/metastasis. Previous studies reported that morphologic changes from cobblestone-like to fibroblast-like occurred in cells undergoing EMT, which is a key process for epithelial cancer cells to survive following detachment from basement membrane and migrate through the extracellular matrix (ECM) to enable cancer cells to “seed” to adjacent tissues or to colonize to distal part of the body through circulation [29,30]. A recent study also demonstrated that both *CLDN3* and *CLDN4* regulated EMT in ovarian cancer cells [31]. Similar to previous study that *CLDN3* has no significant effect on ovarian cancer cell proliferation and cell cycle progression [32], our results also found that ectopic expression of *CLDN3* in HCC cells with absent *CLDN3* expression did not show any change in the cell proliferation rate, cell cycle distribution, and apoptosis *in vitro*. However, we observed a significant inhibitory role of *CLDN3* in liver cancer cell migration and invasion. Moreover, *CLDN3* could significantly inhibit HCC tumor growth *in vivo*. Notably, overexpression of *CLDN3* did not affect cell proliferation *in vitro* but inhibit tumor growth *in vivo*. One possibility is that CLDN3 is a transmembrane protein in the tight junction barrier, which may render its growth inhibition potential *in vivo* in response to extracellular signals originating from the tumor microenvironment. These findings suggested that *CLDN3* may play a critical role in maintenance of normal TJ function to prevent cells from dispersion but not in regulation of pathways that determine the cell fate such as cell cycle or apoptosis.

To better elucidate the invasive and metastatic mechanisms of *CLDN3*, the effect of CLDN3 overexpression or depletion on Wnt signaling pathway and EMT was investigated. Using qPCR analyses, we found the metastasis suppressor role of CLDN3 in HCC cells was associated with the inhibition of Wnt-EMT activity through downregulation of *GSK3B* (encodes GSK3β), *CTNNB1* (encodes β-catenin), *SNAI2* (encodes Slug), and *CDH2* (encodes N-cadherin) by qPCR analyses. Both GSK3β and β-catenin are key molecules of the canonical Wnt pathway implied in many human cancers [33]. Although it remains controversial whether GSK3β is a “tumor suppressor” or “tumor promoter” in regulation of neoplastic transformation and tumor progression, GSK3β expression level was found to be higher in liver tumors than in normal liver tissues in a mouse model of hepatic carcinogenesis [34]. A recent study also showed that both GSK3β and β-catenin expression level were significantly reduced with a concomitant reduction of metastatic capability and expression of Wnt signaling pathway targeted genes in HCC cell line SMMC-7721 subjected to anti-cancer drug treatment [35]. Slug is a critical EMT-inducing transcription factor and its activity was also regulated by canonical Wnt signaling [36]. N-cadherin is a mesenchymal marker which can be induced by Slug [37]. Upregulation of N-cadherin is also associated with a heightened invasive potential in many cancers including HCC [38-41]. Further study by western blot analyses confirmed the changes of β-catenin Slug, N-cadherin in CLDN3 overexpressed or depleted HCC cells. Moreover, the epithelial marker E-cadherin was increased, whereas the mesenchymal marker vimentin was downregulated in CLDN3-overexpressing cells. All of these results postulated that epigenetic silencing of *CLDN3* in HCC might fail to suppress the β-catenin mediated Wnt pathway to promote EMT.

In conclusion, we demonstrated that *CLDN3* is an epigenetically silenced metastasis suppressor gene and plays a crucial role in the development and progression of HCC. *CLDN3* downregulation was an independent prognostic factor for poor survival of HCC. A better understanding of the molecular mechanism of *CLDN3* in inhibiting liver cancer cell metastasis may lead to a more effective management of HCC patients with the inactivation of *CLDN3*.

## METHODS

### Cell lines and cell culture

Ten human HCC cell lines (HepG2, Hep3B, Huh1, Huh6, Huh7, SNU398, SNU449, PLC5, Bel7404, and MHCC97H) were used in this study. Cell lines were maintained in Dulbecco's modified Eagle's medium supplemented with 10% fetal bovine serum (FBS) and 1% penicillin-streptomycin.

### Patients and tissue samples

Two cohorts of clinical HCC tissue samples were investigated in this study. Cohort 1 includes 52 pairs of primary HCC tumors and their adjacent nontumorous tissues, which were collected immediately after surgical resection prior to any other therapeutic intervention at the Third Affiliated Hospital of Sun Yat-Sen University (Guangzhou, China). All samples were confirmed by histology. Informed consent was given by all of the patients. The study protocol was approved by the Clinical Research Ethics Committee of the Sun Yat-Sen University of Medical Sciences. Cohort 2 includes cDNA samples synthesized from 114 pairs of primary HCC tumors and their adjacent nontumorous tissues, which were kindly provided by Prof. XY Guan at the University of Hong Kong [42]. Human normal liver tissue RNA samples were purchased commercially (Stratagene, La Jolla, CA). Clinical data of Cohort 2 patients included in this study are detailed in Supplementary Table 1.

### RT-PCR and real-time quantitative PCR (qPCR)

Total RNA was extracted by Direct-zol RNA MiniPrep kit (Zymo Research, Irvine, CA) from cell pellets or tissues. cDNA was synthesised by MultiScribe Reverse Transcriptase (ABI, Calsbad, CA). RT-PCR was performed with TaKaRa Taq Hot Start Version kit (Takara, Japan). Q-PCR was performed with Power SYBR Green PCR Master Mix reagent (ABI). 10ng cDNA template and 3pmol of primer pairs were applied in each 10μl PCR reaction. All qPCR primers used in this study were acquired from online primer database (medgen.ugent.be) and purchased commercially (Invitrogen, Calsbad, CA).

### 5-aza-2′-deoxycytidine (5-Aza) treatment

1×10^7^ cells of each HCC cell line were seeded into 100mm culture dishes and incubated overnight. 5-Aza (Sigma, St. Louis, MO) was then added into the cell culture at a final concentration of 2μM and incubated for 96h. After 5-Aza treatment, the cells were harvested for RNA extraction and further analysis.

### Bisulfite treatment and promoter methylation analysis

Genomic DNA was extracted by QIAamp DNA Mini Kit (Qiagen, Valencia, CA) and 1 mg of DNA was bisulfite-modified by EZ DNA Methylation-Gold Kit (Zymo Research) following the manufacturer's instructions. The bisulfite-modified DNA was then amplified by methylation-specific PCR (MSP) using primer pairs that specifically amplify either methylated or unmethylated sequences of the *CLDN3* gene. Bisulfite genomic sequencing (BGS) was performed to assess the methylation levels of 9 CpG sites spanning from -117 to -2 of the *CLDN3* promoter region in HCC cell lines. Nucleotide sequences of the primers were listed in Supplementary Table 2.

### *CLDN3* cloning and lentivirus transduction

The full-length *CLDN3* cDNA was obtained by RT-PCR using normal human liver cDNA as template. The PCR product was cloned into pcDNA3.1/V5-His-TOPO TA vector (Invitrogen) and the authenticity of the sequence was verified by sequencing (BGI, Shenzhen, China). The pCDNA3.1-CLDN3 plasmid was then sub-cloned into lentiviral eukaryotic expression vector pLVX-ZsGreen1-PGK-Puro by commercially available service (Genomeditech, Shanghai, China) and the lentivirus particles were packaged by GM easy lentivirus packaging kit (Genomeditech) in 293FT cells and harvested after 48h incubation by following the manufacturer's instructions. The lentiviral particles containing either empty vector or CLDN3 construct were transducted into HCC cell lines and the cells were then subjected to puromycin (2μg/ml) selection for at least 2 weeks to establish stable CLDN3-expressing clones.

### RNA Interference

Small interfering RNA (siRNA) (100 nM) against *CLDN3* or a scrambled sequence (RiboBio Co. Ltd. Guangzhou, China) was transfected into cells in 6-well plates using jetPRIME transfection reagent (Polyplus-Transfection, Illkirch-Graffenstaden, France) according to the manufacturer's instructions. At 48h after transfection, the effects of gene silencing were measured via qPCR and western blot analysis.

### Foci formation assay

1×10^3^ of CLDN3-transfected or empty vector-transfected HCC cells were seeded in 6-well plates and selected with puromycin (2μg/ml) for 7 days. Surviving colonies were then visualized by 1% crystal violet staining and colonies with no less than 50 cells/colony were counted.

### Cell proliferation assay

Cell proliferation was determined by xCELLigence RTCA DP system (Roche, Kaiseraugst, Switzerland). Briefly, 5×10^3^ HCC cells were seeded into each well of the 16-well E-plate supplied by 200μl DMEM culture medium in triplicates. The xCELLigence system was then used to monitor the cell growth rate continuously for 72h.

### Cell cycle and apoptosis analysis

Cell cycle distribution and apoptosis were examined by flow cytometry. For cell cycle analysis, after 12h of synchronization by serum starvation, the CLDN3-transfected HCC cells were incubated with 10% fetal bovine serum (FBS) for 24h. Cells were fixed in 70% ethanol and stained with 50 μg/mL propidium iodide (BD Pharmingen, San Jose, CA). The cells were then sorted by FACSCalibur (BD Biosciences, San Jose, CA) and cell-cycle profiles were analyzed by WinMDI v. 2.9 software (Scripps Research Institute, La Jolla, CA). Apoptosis was assessed by flow cytometry after staining with FITC conjugated Annexin V and 7-amino-actinomycin (7-AAD) following the manufacturer's instruction (BD Biosciences).

### Migration assay and invasion assay

For cell migration assay, cells were seeded into 24-well plate (2×10^5^/well) and incubate overnight. After starving in serum free medium for 24h, the cell layer was wounded using a sterile tip and the medium was then replaced by DMEM containing 10% FBS. The crosses in the wells were photographed by microscopy at time point 0 hour and 24 hours. The percentages of open wound area were calculated using TScratch software [43] kindly provided by Dr. Johnny Koon at the Chinese University of Hong Kong. The experiment was performed in triplicate. For invasion assay, cells were starved with serum free medium for 24h before the assay. Cells (1×10^5^) were suspended in 0.5ml serum-free medium and loaded on the upper compartment of invasion chamber coated with Matrigel (BD Biosciences). The lower compartment was filled with complete medium as chemoattractant. After 24h, invasive cells were fixed, stained, and counted under a microscope. Triplicate independent experiments were performed.

### Immunohistochemistry (IHC)

IHC staining was performed using the standard streptavidin-biotin-peroxidase complex method. Briefly, paraffin sections of HCC tissues were deparaffinized, blocked with 10% normal goat serum for 10min, and incubated with anti-CLDN3 polyclonal antibody (Invitrogen, 1:800 dilution) overnight at 4°C. The tissue section was then incubated with biotinylated goat anti-rabbit immunoglobulin at a concentration of 1:75 at 37°C for 30min. The status of CLDN3 expression was assessed by two independent investigators without prior knowledge of clinicopathologic data. The extent of CLDN3 staining was scored manually by assigning the percentage of positive tumor cells (0, none; 1, <40% of positive staining cells; 2, 40-70% of positive staining cells; 3, >70% of positive staining cells).

### *In vivo* tumorigenicity

1×10^7^ of stable CLDN3-overexpression cells and control cells were injected subcutaneously into the dorsal right flank of 6-week-old male Balb/c nude mice (n=5 per group), respectively. Tumor diameter was measured every 3 days from the 5th day after inoculation for 17 days. The tumor volume was calculated by the formula V = 0.5×L×W^2^. All experimentations on animals were approved by the Animal Experimentation Ethics Committee of the Chinese University of Hong Kong.

### RT2 profiler PCR array

Total RNA extraction was performed using the Direct-zol RNA MiniPrep kit as described above. The first-strand cDNA synthesis was performed using a RT^2^ First-Strand cDNA Synthesis kit (Qiagen) and 1000 ng of total RNA was processed for the human WNT signaling pathway PCR array (SABiosciences, PAHS-043Z, Frederick, MD). Alterations in mRNA transcript levels between HepG2-CLDN3 and HepG2-Ctl groups were initially analyzed using SABiosciences webportal software (http://www.sabiosciences.com/pcrarraydataanalysis.php). Fold changes and *P* values were calculated using Student's t-test. A *P* value<0.05 with a fold change greater than 3.0 were considered to be a significant dysregulation.

### Western blot

Total protein was extracted and the concentrations were determined by Bradford Protein Assay (Bio-Rad, Hercules, CA). 30μg of protein from each sample was separated on 8~12% SDS-PAGE and transferred onto polyvinylidene fluoride membrane (Bio-Rad). Blots were detected by incubation with antibodies to CLDN3 (Invitrogen), total/active β-catenin, E-cadherin, Vimentin, N-cadherin, and GAPDH (Cell Signaling, Danvers, MA).

### Statistics

Statistical analysis was performed with the SPSS standard version 16.0 (Chicago, IL) or GraphPad Prism 5 (GraphPad Software, La Jolla, CA). Results expressed as mean ± SD were analyzed using the Student *t* test. The difference of CLDN3 expression between tumor and adjacent normal tissues were compared using the Mann-Whitney U test. Correlation between CLDN3 expression and clinicopathological features were determined by Pearson's Chi-square test. Overall survival curves was assessed by the Kaplan-Meier method and compared by the log-rank test. Relative risks of cancer-related death associated with *CLDN3* expression status were estimated by univariate analyses. Multivariate survival analysis was carried out on all parameters that were found to be significant on univariate level using the Cox regression model. P value less than 0.05 was considered statistically significant.

## SUPPLEMENTARY-MATERIAL FIGURES AND TABLES


