# Supplementary material for: CLDN3 inhibits cancer aggressiveness via Wnt-EMT signaling and is a potential prognostic biomarker for hepatocellular carcinoma
Source: Oncotarget. 2014 Jul 31;5(17):7663–76. doi: 10.18632/oncotarget.2288 (PMC4202152; doi:10.18632/oncotarget.2288)
Supplement: Supplementary file 1 [file oncotarget-05-7663-s001.pdf]

CLDN3 inhibits cancer aggressiveness via Wnt-EMT signaling and is a potential prognostic biomarker for hepatocellular carcinoma

Supplementary Material

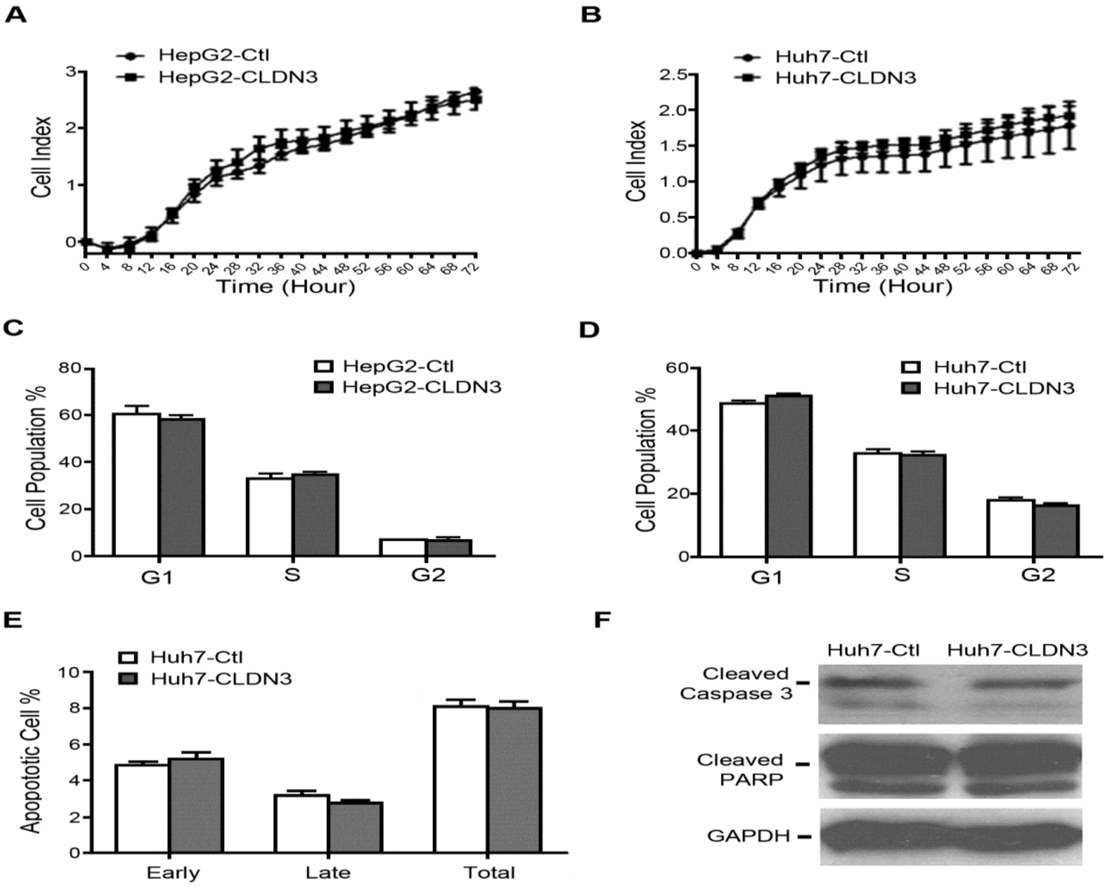

Fig. S1: *In vitro* functional role of *CLDN3* in HCC cells. No obvious difference was observed between *CLDN3*- and empty vector-transfected HepG2 and Huh7 cells by cell proliferation (**A**, **B**) and cell cycle (**C**, **D**) analyses. (**E**) Cell apoptosis analyses showed the percentage of apoptotic cells did not change between *CLDN3*- and empty vector-transfected Huh7 cells. (**F**) Expressions of cleaved caspase 3 and cleaved PARP were compared by Western blotting analysis between *CLDN3*- and empty vector-transfected Huh7 cells. GAPDH was used as loading control.

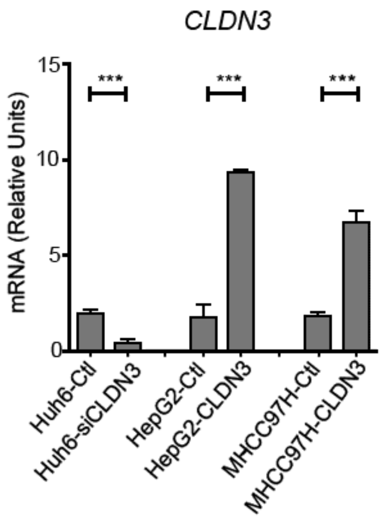

Fig. S2: Ectopic expression of *CLDN3* in HCC cell lines (HepG2 and MHCC97H) and knockdown of *CLDN3* in HCC cell line Huh6 were confirmed by qPCR. ***P<0.001.

Supplementary Table 1: Clinicopathological features and *CLDN3* expression in 114 HCC patients

Case#	CLDN3[a]	Gender[b]	Age	HBsAg[c]	AFP	Tumor Size	Cirrhosis[d]	Differ[e]	TNM[f]	Met[g]	Censor[h]	Surviral
1	0	2	50	2	10,710.00	10.00	0	3	2	0	1	96.30
2	1	2	37	2	17,631.00	9.20	0	3	1	0	1	91.10
3	0	2	28	2	402.10	3.50	0	1	1	1	2	78.10
4	1	2	30	2	10.32	6.00	0	2	1	1	1	11.50
5	1	2	47	2	2,528.00	6.00	0	1	1	1	1	83.60
6	0	2	66	1	14.73	3.50	0	2	1	0	1	84.50
7	1	1	45	2	2.36	19.00	0	3	1	0	1	30.40
8	0	2	52	2	2.44	17.00	1	2	2	0	1	48.10
9	0	2	59	2	1,966.00	9.00	0	3	1	0	1	32.20
10	0	2	35	2	16.07	5.50	0	1	2	0	1	54.00
11	0	2	58	2	21.54	4.50	1	1	1	0	2	35.00
12	0	2	37	2	184.70	5.10	0	2	1	1	1	57.20
13	1	2	67	2	7.98	10.00	0	-	1	1	1	78.30
14	0	1	52	2	414.10	3.00	0	3	1	0	1	46.60
15	0	2	39	2	4,769.00	3.50	-	3	1	0	1	72.70
16	1	2	46	2	1,565.00	12.00	0	2	1	1	2	66.20
17	0	2	43	2	392.50	9.00	1	3	1	0	1	57.40
18	0	2	70	2	36.73	5.00	1	3	2	1	1	9.00
19	1	2	33	2	43.38	9.00	0	1	2	1	1	8.20
20	0	1	52	2	25,150.00	8.00	0	3	1	1	1	6.20
21	0	2	58	2	504.50	3.50	1	3	1	0	1	75.60
22	1	2	38	2	2,416.00	4.00	0	2	1	0	1	75.90
23	1	2	55	2	310.20	2.50	0	2	1	1	1	59.90
24	1	2	40	2	4.21	6.00	0	1	1	1	1	73.40
25	1	2	45	2	2,440.00	12.00	0	3	2	1	1	49.60
26	0	2	65	2	-	10.00	0	1	1	0	1	52.10
27	0	2	65	2	340.40	4.00	0	3	1	0	2	14.00
28	0	2	42	2	19,618.00	3.50	1	2	1	1	2	20.40
29	0	2	50	-	-	6.00	0	-	2	0	2	6.00
30	0	1	39	1	1.91	6.50	0	3	1	0	2	40.80
31	0	2	55	2	37.20	12.00	0	3	1	1	2	16.60
32	0	2	54	2	214.30	5.00	1	3	1	1	1	41.00
33	0	1	31	2	7,162.00	7.00	1	1	1	1	2	7.80
34	0	2	52	2	121,000.00	12.00	0	3	1	1	2	3.30

35	0	1	39	2	5,449.80	4.50	1	3	2	1	2	12.30
36	1	1	63	2	20,394.00	5.00	1	2	1	1	1	43.80
37	0	2	52	2	5.04	3.50	0	3	1	0	2	2.40
38	0	2	47	2	837.10	3.00	0	3	1	1	2	25.90
39	0	2	52	2	43.30	3.00	1	2	1	1	1	40.70
40	1	2	50	2	6.12	4.50	0	3	2	0	1	31.90
41	0	2	43	2	19,582.00	5.00	0	3	1	1	2	16.40
42	0	2	54	2	104,826.00	14.00	0	2	2	1	2	5.00
43	0	2	58	2	361.20	2.00	1	2	1	1	2	35.00
44	0	2	62	2	3.30	8.00	0	1	1	1	2	40.20
45	0	2	55	2	50,979.00	7.00	0	2	1	1	2	51.50
46	0	2	17	2	3,177.00	3.70	0	3	2	0	2	3.80
47	0	1	47	2	34.02	11.00	1	3	2	1	2	6.30
48	0	2	31	2	121,000.00	11.00	0	3	1	0	1	23.10
49	1	2	50	2	2.04	6.00	0	2	1	1	1	59.80
50	0	2	49	2	4.17	12.00	1	-	1	0	1	28.40
51	1	2	50	2	76.85	9.00	1	3	2	1	2	33.00
52	1	1	33	1	14,629.00	6.50	0	3	2	1	2	6.50
53	0	2	32	2	105.10	8.00	1	2	1	1	1	16.80
54	0	2	43	2	24,250.00	17.00	0	3	1	1	1	23.10
55	0	2	41	2	167.80	5.00	1	3	1	1	2	32.10
56	1	2	55	2	83.42	4.00	0	3	1	1	1	35.30
57	0	2	43	2	231.70	8.00	0	3	1	1	2	11.20
58	0	1	53	1	39.68	8.50	0	3	2	0	-	-
59	0	2	58	1	3.99	12.00	0	1	1	0	1	32.90
60	0	2	60	2	6.35	5.00	1	2	1	0	1	14.70
61	0	2	52	2	478.50	6.00	0	2	2	0	1	36.80
62	0	2	30	2	3,904.00	3.00	0	3	1	1	1	3.30
63	0	1	60	1	2,324.00	7.00	0	3	2	1	1	6.80
64	1	1	29	2	5,414.00	2.50	0	3	1	0	1	31.20
65	0	2	39	2	6,535.00	10.00	1	2	1	1	1	5.10
66	1	2	70	2	121,000.00	12.00	1	3	1	0	2	2.10
67	0	2	58	2	11.42	9.00	0	2	1	0	1	24.90
68	0	2	52	2	5.42	6.50	0	2	1	1	2	18.20
69	0	1	47	2	8,406.00	10.00	0	2	1	0	1	36.90
70	0	1	43	1	0.95	15.00	0	1	1	0	1	34.30
71	0	2	63	2	7,301.00	12.00	0	3	2	1	2	9.80
72	1	2	52	2	2.06	12.00	0	2	1	0	1	52.00

73	0	2	35	2	87.47	9.00	1	1	1	1	2	39.60
74	0	2	48	2	15.60	5.00	0	2	2	1	1	20.20
75	0	2	58	2	4.31	2.50	0	2	1	1	1	50.80
76	0	2	38	-	-	-	-	-	-	0	-	-
77	0	2	37	2	4.22	7.00	0	1	1	0	1	50.40
78	0	2	36	1	240.40	4.00	0	2	1	0	1	50.30
79	1	2	35	2	1,981.00	5.00	1	2	1	0	1	35.20
80	0	1	33	2	47,205.00	8.50	-	2	1	0	1	47.20
81	0	2	59	2	3,679.00	5.00	0	2	1	1	1	18.60
82	0	1	31	2	6.11	7.50	0	2	1	1	1	48.80
83	1	2	32	2	39.67	12.00	0	3	1	0	1	47.30
84	0	2	31	2	302.70	6.00	0	3	1	1	1	20.60
85	0	2	51	2	440.00	9.50	0	3	1	1	1	46.00
86	0	2	67	2	2.06	3.00	0	1	1	0	1	45.70
87	1	2	45	2	75.40	12.00	1	2	2	1	1	46.70
88	0	2	64	2	8.95	2.60	1	2	1	0	1	43.80
89	1	2	45	2	11.59	5.00	1	2	1	1	1	43.80
90	0	1	54	2	1,806.00	5.00	0	2	1	0	1	25.10
91	0	2	46	2	483.10	2.50	0	1	1	0	1	43.00
92	0	2	50	2	5.72	8.00	0	3	1	0	1	29.30
93	1	2	57	2	26.38	2.00	1	2	1	1	1	44.80
94	0	2	60	1	1.66	4.50	0	2	1	0	1	44.80
95	0	2	53	2	10,677.00	4.50	1	2	1	1	1	44.60
96	0	2	34	-	-	-	-	-	-	-	2	-
97	0	1	46	1	7.02	6.00	0	3	-	-	2	8.07
98	0	2	32	2	-	1.30	1	2	-	1	2	14.87
99	1	2	56	1	29.16	9.50	0	3	-	0	1	41.67
100	0	2	66	1	2.52	9.00	0	-	-	-	-	-
101	0	1	21	1	7,034.00	5.00	0	3	-	1	2	19.13
102	0	2	58	1	6,846.00	4.50	0	2	-	0	1	39.90
103	0	2	53	-	-	-	-	-	-	0	1	-
104	0	2	67	1	849.00	13.00	0	3	-	0	1	32.27
105	0	1	68	2	2,374.00	5.00	0	3	-	0	1	31.63
106	0	2	74	2	630.30	4.00	1	2	-	0	1	39.00
107	1	2	43	-	121,000.00	14.00	1	3	-	0	1	38.00
108	0	2	39	2	3.24	2.00	1	2	-	0	1	18.67
109	0	2	72	1	5.61	5.50	0	2	-	0	1	34.80
110	0	2	47	1	3,515.00	5.00	1	2	-	0	1	31.47

111	0	1	21	2	8,250.00	12.00	0	2	-	1	1	5.47
112	0	2	36	1	0.96	3.50	0	2	-	0	1	10.30
113	0	2	70	-	-	-	-	-	-	0	2	6.27
114	0	2	36	2	8.06	3.00	0	2	-	0	1	33.90

[a]CLDN3 expression detected by qPCR: 0=down-regulation, 1=normal expression;

[b]Gender: 1=female, 2=male;

[c]HBsAg: 1=negative, 2=positive;

[d]Cirrhosis: 0=absent, 1=present;

[e]Differentiation: 1=well differentiated, 2=moderately differentiated, 3=poorly differentiated;

[f]TNM stage: 1=I-II stage, 2=III-IV stage;

[g]Recurrence or metastasis: 0=absent, 1=present;

[h]Censor: 2=dead at the end of the follow-up, 1=still alive at the end of the follow-up.

Supplementary Table 2: List of PCR primers for expression, methylation, and bisulfite sequencing analyses.

Primers	Sequence (5'-3')	Product Length (bp)
RT-PCR		
CLDN3-F	CAACACCATTATCCGGGACT	168
CLDN3-R	TTGGTGGCCGTGTACTTCTT	
ACTB-F	GTCTTCCCCTCCATCGTG	113
ACTB-R	AGGGTGAGGATGCCTCTCTT	
MSP		
CLDN3 MSPm-F	TTCGAGTAGAGAGGGATAAGGATC	171
CLDN3 MSPm-R	CTCCAAAACCTAAAAACAATAACGTA	
CLDN3 MSPu-F	TTTTGAGTAGAGAGGGATAAGGATT	172
CLDN3 MSPu-R	CTCCAAAACCTAAAAACAATAACATA	
BGS		
CLDN3BGS-F	GAGTAGAGAGGGATAAGGAT	290
CLDN3 BGS-R	CCTACACCTACCTATAACTTTATAAAC	
